# Geochemical Evidence of Metal-Driven Anaerobic Oxidation of Methane in the Shenhu Area, the South China Sea

**DOI:** 10.3390/ijerph16193559

**Published:** 2019-09-23

**Authors:** Rui Xie, Daidai Wu, Jie Liu, Tiantian Sun, Lihua Liu, Nengyou Wu

**Affiliations:** 1 Key Laboratory of Gas Hydrate, Guangzhou Institute of Energy Conversion, Chinese Academy of Sciences, Guangzhou 510640, China; xierui@ms.giec.ac.cn (R.X.); liujie1@ms.giec.ac.cn (J.L.); sutt@ms.giec.ac.cn (T.S.); liulh@ms.giec.ac.cn (L.L.); 2 Institution of South China Sea Ecology and Environmental Engineering, Chinese Academy of Sciences, Guangzhou 510301, China; 3 University of Chinese Academy of Sciences, Beijing 100049, China; 4 Laboratory for Marine Mineral Resources, Pilot National Laboratory for Marine Sciences and Technology (Qingdao), Qingdao 266071, China; wuny@ms.giec.ac.cn; 5 Key Laboratory of Gas Hydrate, Ministry of Natural Resources, Qingdao Institute of Marine Geology, Qingdao 266071, China

**Keywords:** sulfate-drive methane anaerobic oxidation, metal-drive methane anaerobic oxidation, gas hydrate, Shenhu area

## Abstract

Anaerobic oxidation of methane (AOM) is a common biochemical process in the ocean and it plays an important role in global climate change, elemental circulation, and atmospheric evolution over geological time. In this paper, we analyzed of δ^34^S, Fe, Mn, Ca/Ti, and Sr/Ti ratios, and the date of carbon and sulfur from the site SH3 of Shenhu area. Result showed that (1) 0–6 mbsf (meter blow the sea floor) was mainly affected by OSR (anaerobic oxidation of organic matters) and 7–15 mbsf was a paleo-SMTZ (sulfate–methane transition zone) position. The modern SMTZ was mainly distributed at 19–25 mbsf. The barium sulfate precipitation above the modern SMTZ indicating that the current methane leakage was stable and lasted longer during geological history. (2) By studying the change of magnetic and the different carbonate minerals, results showed that there were two AOM stages. During the early stage, Fe^2+^ were mainly produced by sulfide abiotic reductive dissolution. During the later stage, Fe^2+^ were mainly produced by the metal-AOM. (3) Study of the mineral characteristics of the paleo-SMTZ and the modern SMTZ showed that the modern SMTZ carbonate minerals were mainly low-Mg calcite and aragonite, while the paleo-SMTZ carbon minerals were mainly high Mg minerals. The reason for this difference is that the modern SMTZ layer was only experienced the first stage of anaerobic oxidation of methane. In the paleo-SMTZ layer, it has experienced two stage of anaerobic oxidation of methane. During the last stage of metal-AOM, the low Mg carbonate minerals were converted into high Mg carbonate minerals. This research confirms the presence of metal-driven methane anaerobic oxidation at the bottom of sulfate-driven methane anaerobic oxidation and during the metal-driven methane anaerobic oxidation, methane and metal oxides or hydroxides would couple to convert the in situ metal oxides or hydroxides into metal ions, meanwhile the phosphorus adsorbed on the surface of the metal oxides is released into adjacent pore water, and convert to new P-bearing minerals under suitable conditions.

## 1. Introduction

Methane seepage is a global phenomenon that occurs at the continental margins [1,2,3,4]. Large quantities of methane released from sediments are considered to be a important factor influencing global climate change [2,5,6]. The anaerobic oxidation of methane has an important influence on seepage and release of methane [7,8,9,10]. In the marine environment, AOM is often coupled with sulfate, methanogens, and sulfate-reducing bacteria (SRB) to form SO_4_^2−^-AOM [11,12,13,14]. The chemical equation for this reaction is

CH_4_ + SO_4_^2−^ → HS^−^ + HCO_3_^−^ + H_2_O (1)

However, in the process of methane escape, almost 90% is consumed by a biochemical process called microbial anaerobic oxidation of methane (AOM) [6,15]. Much research has been conducted on AOM in the Shenhu area of the northern South China Sea [16,17,18]. In the hydrate drilling area of Shenhu, the sulfate–methane interface (SMI) is about 20 mbsf [19,20,21,22,23,24,25]. The steep gradient of sulfate and methane within the near-surface is a reliable indicator for The AOM [26,27]. Most of the methane produced in the continental shelf and slope sediment diffuses upwards along this gradient to meet sulfate in the sulfate–methane transition zone (SMTZ), where the methane was quantitatively oxidized by anaerobic methanotrophic archaea (ANME) [28]. During the AOM, the ratio of sulfate and methane into the SMTZ is often 1.4:1 [19]. The 40% excess sulfate is used for organic clastic sulfate reduction by the oxidation of organic matter buried into the SMTZ [29,30]. At the SMTZ, reducing conditions can result in reductive dissolution of magnetic Fe-oxides and alteration of the initial sediment composition and magnetic properties due to the replacement of magnetic Fe-oxides by paramagnetic authigenic Fe-sulfides [31,32]. During this process, the surface-adsorbed P (referred to here as P_Fe_) from metal oxides and hydroxides are released into the pore water. These unsteady P are re-adsorbed and precipitated, and some form self-generated phosphate minerals. The SMTZ is also the sedimentary interval where methane-driven autogenous carbonate (MDAC) precipitates. AOM (Equation (1)) elevates porewater alkalinity [26,27], and thus promotes the precipitation of Ca(Mg/Sr)CO_3_ (Equation (2))

(2) $$2{\mathrm{HCO}}_{3}^{-}+{\mathrm{Ca}}^{2+}\left(\frac{{\mathrm{Mg}}^{2+}}{{\mathrm{Sr}}^{2+}}\right)\to \mathrm{Ca}\left(\mathrm{Mg}/\mathrm{Sr}\right){\mathrm{CO}}_{3}+{\mathrm{CO}}_{2}+H_{2}O$$

In the carbonate formed by the SMTZ layer, the value of δ^13^C in the carbonate is generally consistent with the value of δ^13^C in the organic gas. Carbonates with δ^13^C values less than −30‰ VPDB are generally consistent with carbon sourced from gas hydrate, and MDAC is often composed of aragonite, high-Mg calcite [20]. MDAC provides direct geological evidence of AOM and methane seepage in the sedimentary record [28,29,30,31]. Studies of the morphological distribution of pyrite have shown that the sulfate methane transition zone (SMTZ) where AOM occurs is mainly made up of three forms of pyrite: framboids, framboids with overgrowths, and euhedral crystals [27]. Among these, framboidal pyrite is mainly distributed in the area where OSR occurs. The framboids with overgrowths and euhedral crystal pyrite are mainly found in the area where SO_4_^2−^-AOM occurs [32]. Some scholars have also studied the role of AOM from the perspective of foraminifera. Studies of pore water and pyrite value of δ^34^S in the northern South China Sea have found that the SO_4_^2−^-AOM results in a positive deviation of δ^34^S [33]. During SO_4_^2−^-AOM sulfate is usually consumed under semi-closed or closed conditions, reflected in ^34^S-enriched sulfide minerals [31,32,33]. Often, when methane is present, the majority of sulfate available in marine pore fluids is reduced through SO_4_^2−^-AOM. The work on the isotopic composition of sulfur bearing minerals enclosed in sediments and carbonates from cold seeps of south China sea increasing the understanding of biogeochemical sulfur cycling in seep settings. Resluts showed an extreme variability in δ^34^S values ranging from −51.3‰ to 114.8‰ [34]. Under the conditions typical of the sulfate methane transition zone (SMTZ), the sulfate available for SO_4_^2−^-AOM is ^34^S-enriched, which causes the δ^34^S values of SO_4_^2−^-AOM-derived pyrite to be very positive, typically higher than 21‰ [35]. In methane-rich environments, the ^34^S-^32^S fractionation of SO_4_^2−^-AOM is usually smaller than 40‰. Recent studies have shown that microbial AOM can also be coupled with metal oxides and hydroxides to form metal-AOM, mainly including Fe(III) and Mn(IV) reactions [28,36]. The specific reactions are

CH_4_ + 4MnO_2_ + 7H^+^ → HCO_3_^−^ + 4Mn^2+^ + 5H_2_O (3)

CH_4_ + 8Fe(OH)_3_ + 15H^+^ → HCO_3_^−^ + 8Fe^2+^ + 21H_2_O (4)

Meanwhile, Fe(III) and Mn(IV) are widely distributed in deposits and methane, and metal-AOM has important impacts on the global methane cycle and related elemental cycles. Previous studies have shown that metal-AOM occurs in both marine and freshwater environments [26,27,28,29,30,31,32,33], and meta-AOM provides more energy than SO_4_^2−^-AOM. Leakage experiments from sediments have shown [37] that sulfate reduction, iron reduction, AOM, and methanogenesis coexist in marine sediments, where the presence of iron oxides greatly accelerates the rate of bacterial sulfate reduction. In the methane-rich sediments of an anaerobic environment, the carbonate phase and phosphate from Fe and Mn may be products of metal-AOM [28] and it has been speculated that metal-AOM processes typically occur in anaerobic, iron enriched, and sulfate-deficient environments [28,31]. The predecessors have done a lot of research on the AOM in the Shenhu area of the northern South China Sea. However, few scholars have studied the relationship between gas hydrate and metal-AOM. In this study, we hope to solve the following scientific questions: (1) Understanding the response of methane anaerobic oxidation to methane leakage. (2) Reveal the effect of metal-AOM on elemental P and Fe cycles. (3) Realize the relationship between sulfate-AOM and metal-AOM.

## 2. Methods

### 2.1. Sampling

The core SH3 sample used in this study was obtained by the Guangzhou marine Geological Survey in 2007 in the Shenhu area of the South China Sea [38], this site is of particular interest due to its high hydrate saturation and thicker hydrate-bearing sediments. Sediment particles are mainly muddy and dominated by silt with clay or sand as a secondary component. In this study, a total of 31 samples were drilled from 0–25 mbsf in core SH3 and the main trace elements were measured separately.

### 2.2. Experimental Methods

The core SH3 sediments were divided into samples on the expedition vessel, pore water was removed, then samples were brought back to the laboratory for freeze-drying before some of the sediments were ground to less than 75 μm.

The major elements from the sediments were determined at the Analytical and Testing Center of the Guangzhou Institute of Energy Conversion, Chinese Academy of Sciences. Thermo ARL ADVANTta IntelliPower™ 2000 X-ray Diffraction Spectrometry (XRF) was used to determine the primary element content. Measured XRF spectral data was converted to elemental and oxide content by UniQuant semi-quantitative analysis software. The trace elements content in the whole rock was analyzed using Agilent 7700e ICP-MS with an inductively coupled plasma mass spectrometer at the Wuhan Shangpu Analysis Technology Co., Ltd. analysis and testing center. Sample processing for ICP-MS analysis was as follows. (1) the 75 μm sample was placed in an oven at 105 °C for 12 h. (2) 50 mg of the powder sample was weighed in a Teflon bomb. (3) 1 mL of high-purity nitric acid and then 1 Ml high-purity hydrofluoric acid was added to the sample. (4) the Teflon sample bomb was inserted into a steel sleeve, tightened, and placed in an oven at 190 °C for more than 24 h. (5) the sample bomb was cooled and placed on a 140 °C hotplate after opening the lid. The mixture evaporated and 1 mL of HNO_3_ was added prior to another evaporate. (6) 1 mL of high-purity nitric acid, 1 mL of MQ water, and 1 mL of the internal standard in (concentration 1 ppm) were added and the Teflon sample was dissolved again. The bomb was inserted into a steel sleeve, tightened, and placed in an oven at 190 °C for more than 12 h. Finally, (7) the solution was transferred to a polyethylene bottle and diluted to 100 g with 2% HNO_3_ for ICP-MS testing.

TOC was measured using a German Heraeus CHN-O Rapid elemental analyzer. Before testing, the appropriate sediments were selected, excess calcium carbonate was removed by adding 10% HCl, and the sample was diluted several times with distilled water before being placed in an oven at 50 °C. TOC test instrument accuracy was greater than 1%. The above experimental process was conducted at the Guangzhou Institute of Geochemistry, Chinese Academy of Sciences. Total sulfur and total carbon tests were performed using a Vario EL cube element analyzer with an accuracy of 0.1%. The rock powder sample was catalytically oxidized in oxygen at a high temperature to burn and decompose, producing a mixture containing C and S gases. These mixed gases were in turn placed into contact with tungsten oxide and copper to convert them to CO_2_ and SO_2_ gases, at which point the sample was separated by a column. A thermal conductivity detector calculated the content of C and S components by comparing the test sample with a standard sample. The above pretreatment and experimental procedures were completed at the Analytical Testing Center of the Guangzhou Institute of Energy Research, Chinese Academy of Sciences.

### 2.3. Source and Measure Method of Sediment Magnetic Data

In this paper, the magnetic data comes from the report of GMGS1: Measuring the Concentration, Nature, and Distribution of Gas Hydrate [38]. Magnetic susceptibility was measured using the Geotek MSCL-S (Standard Multi-Sensor Core Logger). Magnetic susceptibility was measured with an 8-cm-diameter Bartington loop sensor; the error in magnetic susceptibility was ±5% [38].

## 3. Geological Setting

**Figure 1:**
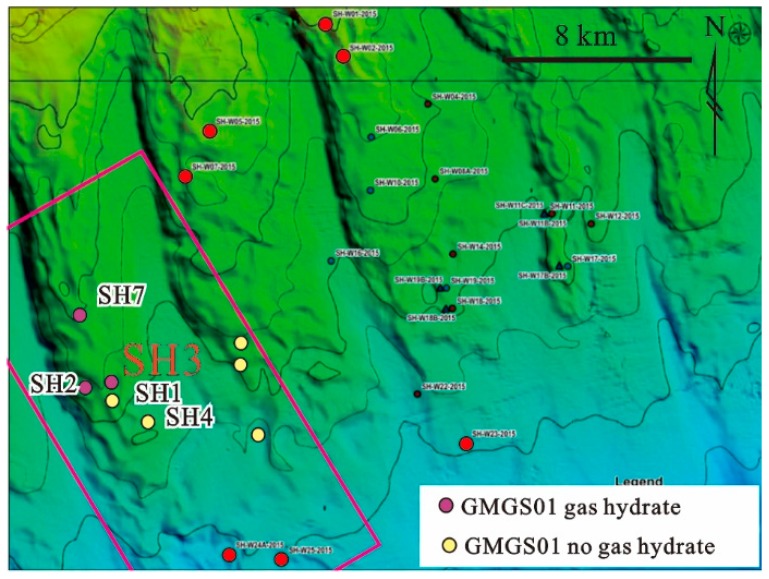
Geological topographic map of the Shenhu area in the northern part of the South China Sea and the location of the core SH3.

The South China Sea is one of the largest marginal seas in the Western Pacific [17]. The northern part of the South China Sea is made up of a passive continental margin with sedimentary sequences ranging from 1000 to 7000 m. Total organic carbon content in organic-rich sediments ranges from 0.46% to 1.90% by weight [39,40]. The Shenhu area is located in the middle of the northern part of the South China Sea (Figure 1). Earthquake data has been used to identify several promising hydrate-bearing areas with strong BSR (bottom-stimulating reflectors) [41]. In the northern part of the South China Sea, higher sedimentation rates provide very good geological conditions for hydrate formation [42]. The area’s rich micro-fractures, folds, and mud diapirs provide migration pathways for methane fluids and the formation of hydrates [43]. Cold spring carbonates have been found in the northern part of the South China Sea including the Shenhu area [29,33,34,35,36,37,38,39,40,41,42,43,44]. In 2007, Guangzhou Marine Geological Survey obtained the first physical sample of hydrate from the Shenhu area. In 2017, Guangzhou Marine Geological Survey successfully carried out hydrate test mining in the area. These findings confirmed the existence of nature gas hydrate in South China and verified that overflowing methane seepage in the northern South China Sea, especially in the Shenhu area, is a very common phenomenon [38].

## 4. Results

### 4.1. Description of Pore Water Changes with Depth

**Figure 2:**
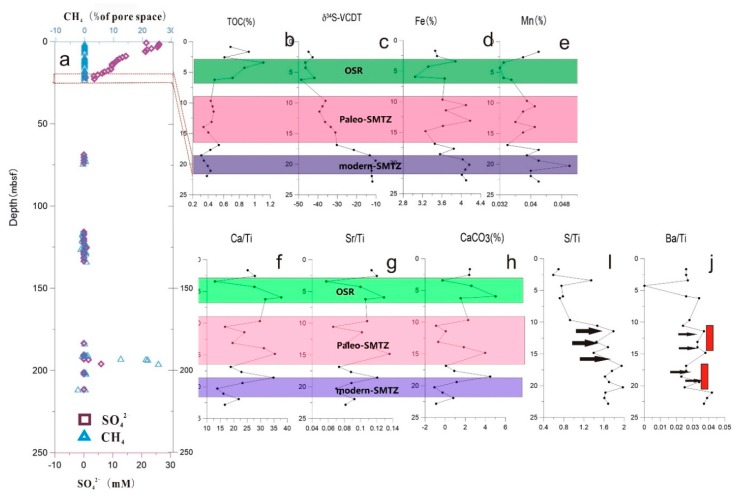
In the picture, the green represents OSR, the red represents paleo-SMTZ and purple represents modern- SMTZ. (**a**) shows the concentration of sulfate and methane in the pore water as a function of depth; (**b**) Total organic carbon content varies with depth; (**c**) δ34S value in sediment changes with depth; (**d**–**h**) Ratios of the main elements Fe, Mn and Ca/Ti, Sr/Ti, S/Ti, Ba/Ti, trends with depth. (**i**,**j**): Black arrows in the Ba/Ti and S/Ti ratios indicate the peaks that were spaced apart: when the S/Ti peak immediately followed the Ba/Ti peak it generally indicated the modern SMTZ interface. Red indicates the concentration of sulfate and methane in the pore water as a function of depth, which shows that the modern SMTZ tended to move vertically.

The change of sulfate content and methane saturation in pore water is an important basis for determining SMTZ. At the core SH3, the content of SO_4_^2−^ in the pore water shows a linear decreasing, and the content at 20 mbsf is close to zero. The content of methane in the pore water is obviously enriched in the 20 mbsf layer, but the content above 20 mbsf is very low (Figure 2a). The content of methane at 20 mbsf is abnormally elevated, which is believed to be caused by the presence of gas hydrate [8]. The change of salinity in the pore water of core SH3 station has two distinct changes. The first stage is the significant decrease of salinity at 6–7.5 mbsf. The second stage is obviously decreasing salinity at 15–17.5 mbsf.

### 4.2. Description of δ^34^S (‰ VCDT)

The value of δ^34^S (‰ VCDT) at different depths measured at core SH3 showed significant difference (Figure 2b). The content of δ^34^S wa relatively stable with the depth increased above 6 mbsf. From 6 mbsf to 16 mbsf, the value of δ^34^S obvious increased, ranging from −49‰ to −30‰ (Table A1). Under 16 mbsf, the value of δ^34^S increase more obvious, ranging from −30‰ to −10‰. Two significant increases in the value of δ^34^S indicate that methane leakage and associated methane anaerobic oxidation may occur twice at the core SH3.

### 4.3. Concentration Profiles of Major and Trace Elements

The content of Fe, Mn presented as the depth increases. Especially at 19–25 mbsf, its content has a significant increase. The concentration of U presented a double-peak pattern as the depth increased. The content of U is significantly increased in the 7–15 mbsf and 19–25 mbsf sediments. Conversely, the content of Mo in sediments shows a single peak with increasing depth. A significant increase at 7–15 mbsf. The content of phosphorus also shows a single peak with depth, but unlike the content of Mo decrease at 19–25 mbsf, which a significant increase at 19–25 mbsf. The ratios of Ca/Ti, Sr/Ti, S/Ti, Ba/Ti and the content of carbonate change trend are similar. They have seen a significant increase in 0–6 mbsf, 7–15 msbf, and 19–25 mbsf. The contents of Mg and Sr in sediments showed opposite trends with depth increasing. At 7–15 mbsf, the content of Mg increase, however, the content of Sr decline. At 19–25 mbsf, the change trend contrary to the trend of 7–15 mbsf. The ratio of Sr/Ca and Mg/Ca change is the same as the change of Mg, Sr. Black arrows in the Ba/Ti and S/Ti ratios indicate the peaks that were spaced apart: when the S/Ti peak immediately followed the Ba/Ti peak, it generally indicated the modern SMTZ interface. Red indicates the bimodal area, which shows that the modern SMTZ tended to move vertically (Figure 2i,j).

## 5. Results and Discussion

### 5.1. Pore Water Sulfate Reduction and Evidence of SO_4_^2−^-AOM

**Figure 3:**
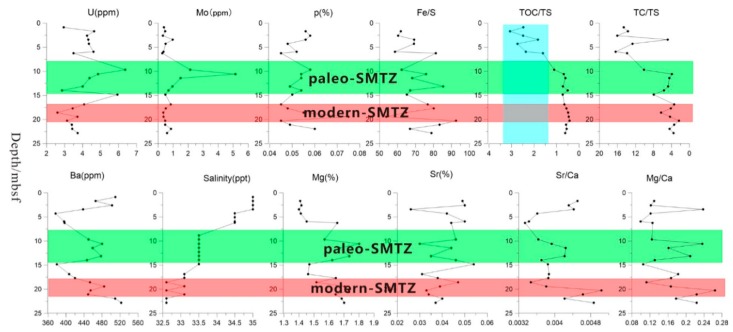
In the picture, the green represents paleo-SMTZ, and the red represents current-SMTZ. Changes in U and Mo coupling indicate that the paleo-SMTZ horizon was a reducing environment and the modern SMTZ was an oxidizing environment; the P element in the sediment had obvious enrichment in the modern SMTZ horizon and the paleo SMTZ layer; a large amount of pore water was released due to metal-AOM, resulting in a significant decrease in pore water in the paleo-SMTZ layer and the modern SMTZ. The Mg/Sr ratio had opposite trends between the paleo-SMTZ and modern SMTZ. This was mainly due to different stages of AOM.

At seeps, most of the methane was consumed at the expense of sulfate via SO_4_^2−^-AOM. SMTZ determination was based on the 1:1 coupling reaction of methane and sulfate concentrations. When the pore water concentrations of sulfate and methane were close to zero, the interface was considered to be the modern SMTZ [16]. Based on the methane and sulphate concentrations (Figure 2a), the modern SMTZ interface should be around 19–25 mbsf. During this process, it can lead to the accumulation of dissolved inorganic carbon and increased alkalinity, which may further trigger the precipitation of authigenic carbonates. At the modern SMTZ for core SH3, we found that the content of calcium carbonate clearly increased (Figure 2h). This also confirmed our assessment of the modern SMTZ position. Previous studies have suggested that elevated Ca/Ti ratios generally result from bioclastics, such as foraminifera shells or shells and methane-driven authigenic carbonate precipitation [16,45]. An increase in the Sr/Ti ratio generally indicates an increase in mineral vermiculite, which is an important methane-driven authigenic carbonate precipitation that occurs when the SMTZ is close to the seabed water–rock interface. At the modern SMTZ (19–25 mbsf) for core SH3, result showed an increase in the Ca/Ti and Sr/Ti ratios, indicating that these could be used as indicators for sulfate-driven methane anaerobic oxidation. Of course, SMTZ was not static and moved with the accumulation of sediments and the continuous supply of methane from below. Therefore, the sediment profile appeared multi-stage carbonate with increases in the of Ca/Ti and Sr/Ti ratios. The anomalies of these elements can help identify the paleo-SMTZ. At the same time, according to previous studies on the Ba/Ti and S/Ti ratios, an increase in the S/Ti ratio immediately after a peak in Ba/Ti generally indicates the SMTZ position [46], while double peaks of Ba indicate vertical movement of the SMTZ (Figure 2f,g). Our analysis of the Ba/Ti and S/Ti ratios at core SH3 also confirmed our determination of the location 7–15 mbsf for the paleo-SMTZ interface (Figure 2f,g). During that SO_4_^2−^-AOM, barium sulfate in the sediments below the SMTZ became unstable and released dissolved Ba^2+^ into the pore water. Barium diffused upward and precipitated as authigenic barium sulfate above the SMTZ [46,47]. The level of authigenic barium sulfate precipitation depended on the upward diffusion of dissolved Ba^2+^ flux and the length of time that the SMTZ remained at that horizon [16,40,41,42,43,44,45,46,47,48]. Results showed that there were two significant enrichments of Ba in core SH3 sediments above the modern SMTZ horizon and the paleo-SMTZ horizon (Figure 3), indicating that precipitation of barium sulfate may have occurred in sediments. The formation of barium sulfate required a stable and longer SMTZ horizon, indicating that the rate of methane seepage at the base of core SH3 was relatively stable and the sedimentation rate was also stable.

As a sink for sulfide in the marine sulfur cycle, sedimentary pyrite is the most abundant sulfur-bearing solid in continental margins. Experiments have shown that pyrite is formed through two major mechanisms: polysulfide and hydrogen sulfide pathways. The hydrogen sulfide pathway for pyrite formation is the reaction between monosulfide and hydrogen sulfide with hydrogen as a byproduct (Equations (5) and (6); [16,28,33,34,35,36,37,38,39,40,41,42,43,44,49]). For both pathways, however, it is believed that δ^34^S pyrite values record the sulfur isotopic composition of the sulfur source in pore water with <1‰ fractionation [50]. In shallow ocean sediments, the environment for pyrite formation is affected by different microbial processes, such as OSR, sulfur disproportionation, and SO_4_^2−^-AOM [28,29,30,31,32,33]. During OSR and SO_4_^2−^-AOM processes, higher HS- concentrations are produced in the pore water and HS- reacts with Fe^2+^ in pore water or sediment to form pyrite [51,52]. The specific reaction is

Fe^2+^ + HS^−^ → FeS + H^+^ (5)

FeS + H_2_S → FeS_2_ + H_2_ (6)

However, δ^34^S values of pyrite from core SH3 were highly variable throughout the core, ranging from −48.6‰ to −9.8‰ (Figure 2). The strong ^34^S depletion of pyrite at 3–6 mbsf (with values as low as −48‰) required a ^34^S depleted pool of dissolved sulfide in the pore water. During OSR, it primarily occurred in the open sulfate system and the preferential microbial turnover of sulfate with the lighter sulfur isotope (^32^S) led to a ^34^S depleted sulfur isotopic composition for the resulting sulfide. Therefore, the sulfide produced by OSR was relatively enriched in ^32^S, so that the formed pyrite had relative low δ^34^S. The δ^34^S values (−48.6‰–−36.3‰) for the negative 0–6 mbsf in the shallow sediment of the Shenhu area indicated that this layer was affected by OSR (Figure 2). However, the value at the 6 mbsf level was −48.6‰. Compared with 21‰ of modern seawater δ^34^S, the isotope fractionation was as high as 78.6‰, which exceeded the experimental maximum value of 70‰ by OSR alone [18,34]. In the shallow process, there may have been other isotope fractionation reactions, such as disproportionation of sulfur.

In contrast, many studies have observed significant ^34^S enrichment in pyrite from methane-rich environments and this pattern has been suggested to reflect pronounced SO_4_^2−^-AOM [13,27,28,29,30,31,32,33,34,50]. Horizons typified by such enrichment have consequently been interpreted to reflect the position of the current and/or paleo-SMTZ [37]. From the sedimentary profile of core SH3, we observed a significant increase in δ^34^S value in the modern SMTZ (Figure 2), and an increase in this (Figure 2) value was also observed at the 7–15 mbsf horizon. Combined with results from Lin et al. (2016) and other analyses of the pyrite content for nearby station SH217, it is believed that there was a paleo–SMTZ interface at the 7–15 mbsf horizon [40].

### 5.2. Evidence of Metal-AOM

At core SH3, sulfate-driven AOM consumed sulfate and methane while producing sulfide and bicarbonate in the pore water; the dissolved sulfide was fixed as Fe monosulfides and under an excess of sulfide, as pyrite. Chen et al. (2013) found that the content of pyrite at station SH3 clearly increased [21]. The ubiquity of pyrite aggregates at the modern SMTZ implies that there was a source of Fe^2+^. The potential sources of Fe^2+^ at the modern SMTZ and below included organic clastic Fe reduction, abiotic reductive dissolution by sulfide, and Fe-AOM [38]. At core SH3, during the modern SMTZ (19–25 mbsf) the sulfate in pore water was consumed by upwelling methane, which formed a relatively low-sulfate environment. Meanwhile, according to the U-Mo covariation system, U and Mo were richer in the anaerobic zone [39]. However, the contents of U and Mo significantly decline at the modern SMTZ of the site SH3. It indicated that the modern SMTZ was oxic zone (Figure 3). Once deposited at the seafloor and buried below the oxic zone, FeOx was partially dissolved by dissimilatory Fe reduction [16,42,43,44], while the adsorbed phosphate was released into the adjacent pore waters. At the modern SMTZ for core SH3, we believe that the dissimilatory reduction of Fe(III) provided a source of metal ions for the formation of a large amount of iron sulfide. Oxygen was gradually consumed during the dissimilatory reduction of Fe(III) and the deposition environment of the modern SMTZ gradually transformed from oxidizing to reducing. During this process, iron oxide was converted into iron sulfide and the dissolved HPO_4_^−^ was transported (either by diffusion and/or advection) to the water column located above the sediments. At the same time, because of the content of U and Mo are defective in core SH3 sediments, the modern SMTZ is in an oxidizing zone, and the reduced iron oxide was easily reoxidized and the released P was re-adsorbed or co-precipitated with freshly precipitated authigenic FeOx at the redox boundary. We therefore found that there was significant P element enrichment in sediments from the modern SMTZ horizon (Figure 3).

**Figure 4:**
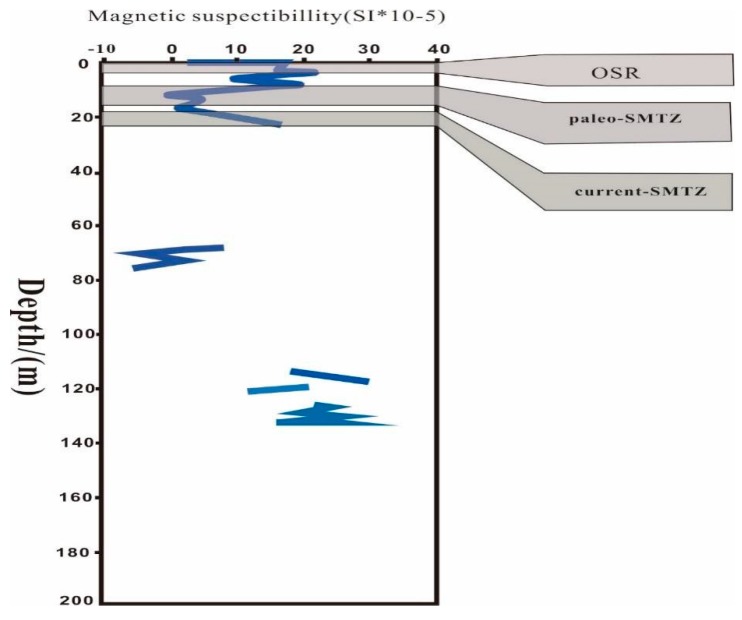
A graph of the change in magnetic properties with depth. The areas marked from top to bottom are OSR, paleo-SMTZ, and current-SMTZ. The magnetic drop in the paleo-SMTZ is obvious, but there was no significant drop in current-SMTZ magnetism. The magnetic data comes from the report of GMGS1: Measuring the Concentration, Nature, and Distribution of Gas Hydrate.

However, it is not enough to rely solely on in situ dissimilatory Fe reduction to produce Fe^2+^. In fact, we know that metal ions play three roles: forming pyrite under suitable conditions, formation of iron-rich carbonate minerals, metal oxides formed by reoxidation, and other mechanisms that are not yet known, respectively. At present, we have discovered apparently enriched pyrite and carbonate minerals in the current horizon. According to the molecular formula of various minerals, it is certainly not enough for the source to rely on the abiotic reductive dissolution by sulfide. The amount of metal ions produced by organic clastic Fe reduction is almost negligible. There must be other sources of metal ions. In 2008, some scholars argued that Fe and Mn might work as ANME eletctron receptors to reduce methane, in a process known as metal-AOM [17,18,51]. Beal (2009) suggested that bioavailable Fe-oxides and potentially reactive Fe-rich silicates below the SMTZ may be available for bacterial Fe reduction coupled to methane oxidation in natural settings [28,52]. At the same time, Beal (2009) proved that when the sulfate concentration is less than 1 mM, the reaction rate of AOM significantly exceeds the reaction rate of sulfate-reducing bacteria (SRB), indicating that metal-AOM occurs under low sulfate concentrations [28]. Sediment incubation experiments have also shown that more poorly reactive Fe minerals (e.g., magnetite and hematite) are also bioavailable and could potentially fuel Fe-AOM [52]. He et al. (2018) calculated the Gibbs free energy of methane oxidation and various environmentally related oxidants. In comparison, metal-AOM produces more energy than SO_4_^2−^-AOM, which is beneficial to the metabolism of microorganisms [8]. Therefore, Fe-AOM certainly has the potential to yield significant quantities of Fe^2+^. During SO_4_^2−^-AOM, however, HS^−^ is generated and reductively dissolves FeO_x_, leading to the precipitation of Fe sulphides (e.g., greigite, mackinawite, pyrite) [52], which can also lead to phosphate enrichment in pore water. The modern SMTZ was in an aerobic zone, which was not conducive to the occurrence of Fe-AOM; however, at the bottom of the modern SMTZ, the U and Mo trends (Figure 3) indicated an anaerobic environment. Therefore, the environment at the bottom of the modern SMTZ was beneficial to the occurrence of metal-AOM. Hsu et al. (2014) reported the formation of vivianite below the SMTZ in the South China Sea sediments, including the intergrowth of Fe sulphides with vivianite micro-aggregates. Liu et al. (2018) found vivianite below the modern SMTZ in the South China Sea Taixinan Basin [30]. The formation of marine vivianite required “a rather unusual set of circumstances” including high organic matter input that rapidly consumed sulphate via bacterial sulphate reduction. The lack of sulfate restricted HS^-^ generation, allowing Fe^2+^ to build up in pore water below the sulphate penetration depth. The Fe^2+^ available to react with phosphate released by organic matter degradation to form vivianite. The formation of marine vivianite indicated the presence of metal-AOM. Prior studies in other parts of the South China Sea have therefore confirmed the existence of metal-AOM at the bottom of the SMTZ, meaning it may have also occurred in the Shenhu area [30]. During metal-AOM, a large amount of ferromagnetic iron oxide was converted into low-magnetic or non-magnetic iron sulfide and metal ions, leading to a significant drop in depositional magnetic properties. Our magnetic analysis of the sediments at core SH3 revealed a significant magnetic drop in the paleo-SMTZ (7–15 mbsf) horizon (Figure 4), indicating metal-AOM occurrence. The changes in sediment magnetic data are consistent with changes in the paleo-SMTZ and current SMTZ positions confirmed by other data. In the SMTZ region, a large number of magnetic minerals (such as magnetite) are converted into paramagnetic or low-magnetic materials (such as pyrite, etc.) so the magnetic in the sediment will drop significantly. However, results did not show a similar drop in the modern SMTZ layer, mainly because it was in an aerobic environment and metal ions were quickly oxidized to form new metal oxides.

**Figure 5:**
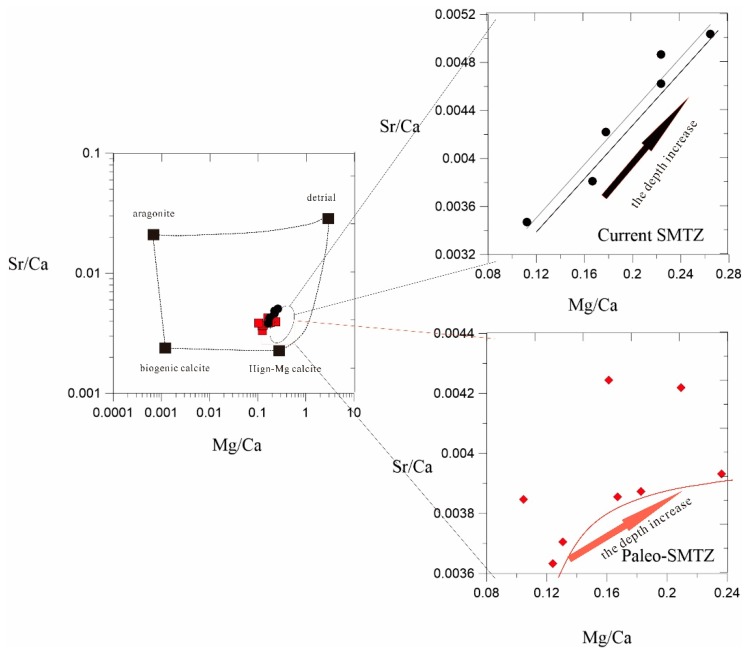
Phase diagrams for Sr/Ca and Mg/Ca in paleo-SMTZ and current-SMTZ deposits were all similar to high-Mg calcite, but the Sr/Ca and Mg/Ca ratios in the current-SMTZ increased with depth. In the early AOM stage, carbonate minerals were mainly from low-calcium calcite and aragonite. The Ca^2+^ in aragonite was easily replaced by Sr^2+^. As the reaction progressed, the Sr in the sediment gradually increased. The Mg/Ca in the paleo-SMTZ gradually increased with depth but Sr/Ca gradually stabilized, mainly because of the small amount of aragonite formed in the early stage was transformed into high Mg by the later metal-AOM. Aragonite content was gradually reduced and Sr in gas sediments gradually stabilized.

Zwicker et al. (2018) proposed that the carbonate that formed in the methane leak zone goes through two stages: the formation of early carbonate minerals is mainly based on low Mg calcite and aragonite. Formation in the later stage is mainly based on high Mg calcite, whereas Zwicker argued that carbonate from high-Mg calcite resulted from the transformation of precursor aragonite—influenced by later-stage seawater circulation or another poorly-understood mechanism [53]. At core SH3, we found that the 7–15 mbsf sediment was dominated by high Mg and low Sr mineral content (Figure 3). At the same time, Ca^2+^ solution in rocks was more easily replaced by smaller cations such as Mg^2+^, while Ca^2+^ in aragonite was preferentially replaced by larger cations like Sr^2+^ [54,55,56,57]. In general, autogenous calcite (especially high-magnesium calcite) was generally rich in Mg, while authigenic aragonite was rich in Sr. At the paleo-SMTZ, the Sr/Ca tended to be stable as depth increased by the Mg content gradually increased (Figure 5). The carbonate that formed in a later stage of AOM was mainly composed of high Mg carbonate minerals. The content of aragonite formed in the early AOM stage was gradually reduced due to the transformation of metal-AOM, so the Sr content in the sediment gradually stabilized [58,59,60,61] (Figure 5). However, the modern SMTZ (17–21 mbsf) was dominated by high Sr and low Mg mineral content, and Mg/Ca and Sr/Ca ratios in the sediment increased. There was also a significant increase in the correlation between Sr/Ca and Mg/Ca (Figure 3). The Mg and Sr content increased with depth, indicating a considerable amount of aragonite was produced and there was continuous replacement of Ca^2+^ in aragonite with Sr^2+^ [62,63,64]. These data imply that carbonate minerals in the paleo-SMTZ underwent two stages, but the modern SMTZ formation of carbonate minerals was still in the early stages, so we believe that in the paleo-SMTZ, early Low-Mg calcite and aragonite were converted to high Mg carbonate minerals by other geological processes. metal-AOM was the dominant geological process. According to the metal-AOM chemical coefficient, large quantities of metal ions, such as iron and manganese, were generated during metal-AOM, which provided a source for metal sulfides in the shallows. At the same time, large amounts of water and heat were generated according to the stoichiometric formula (H_2_O/CH_4_ = 5; H_2_O/CH_4_ = 21) (Table A2). Heat converted Mg calcite into high Mg carbonate minerals and abundant water caused a large drop in pore water salinity (Figure 3). We found that the salinity of the paleo-SMTZ layer at core SH3 decreased significantly and was more obvious that the decline in the modern SMTZ salinity. This indicated a large amount of water production in the paleo-SMTZ layer, as well as the occurrence of metal-AOM.

## 6. Conclusions

**Figure 6:**
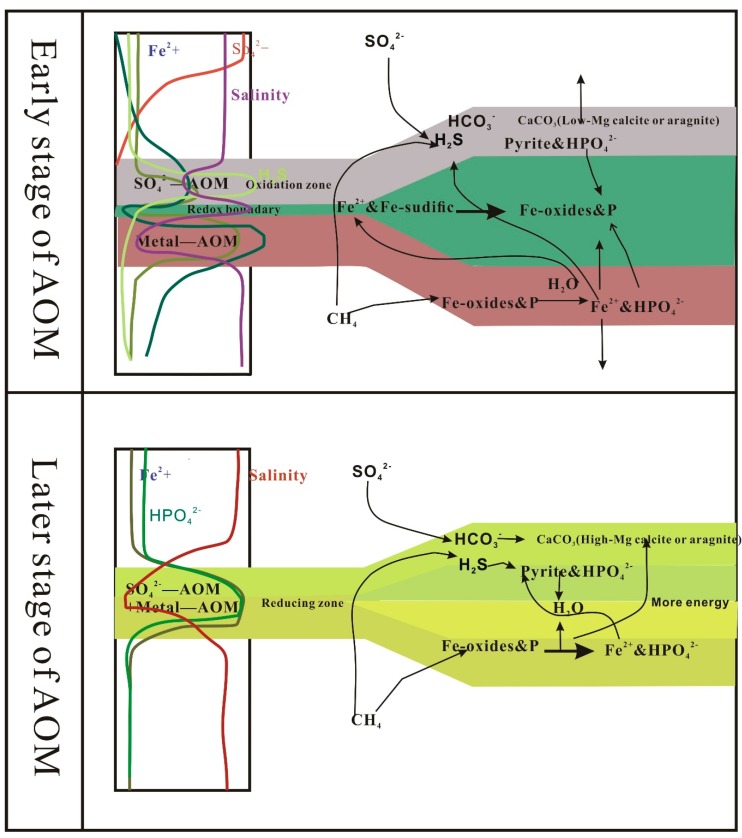
In the early AOM stage of AOM, SO_4_^2−^ and CH_4_ were coupled to form a sulfide environment in the SMTZ layer, and the pore water was enriched in H_2_S. At the same time, the in situ metal oxide was converted into metal sulfide in the SMTZ, creating magnetic properties in the sediment relative decline. Adsorption on the metal oxide surface was also released into the pore water, resulting in the enrichment of a large amount of HPO_4_^−^. The presence of a low sulfate and anoxic environment at the bottom of the SMTZ horizon was dominated by metal-AOM, which converted large amounts of metal oxides and hydroxides into metal ions. Metal ions diffused upwards, some were reoxidized to oxides at the redox boundary, and most continued to move upwards and provided a source of ions for the formation of metal sulfides in the shallow part. A large amount of water was generated during metal AOM. Water caused pore water salinity to decrease, and nutrients such as nitrogen and phosphorus adsorbed on the surface of the metal oxide were also enriched in the pore water and diffused upward, while some were re-adsorbed and precipitated at the redox boundary. In the later AOM stage, the sulfate-driven AOM action and the metal AOM action occurred simultaneously in the SMYZ layer. The two functions converted metal oxide into metal sulfide, the metal ion provided by metal-AOM, and part of the metal formation. Sulfides provided a source of metal ions and some formed in situ an iron-manganese-rich carbonate minerals. Therefore, in the later AOM stage there was a significant decrease in magnetic properties in the sediment. At the same time, due to the large amount of water generated in the later AOM stage, the salinity of the interstitial water also dropped sharply.

In this paper, the change of δ^34^S values, the characteristics of pyrite, and trace element change with the depth showed that 0–6 mbsf was mainly influenced by OSR and 7–15 mbsf was the paleo-SMTZ position at the site SH3. The modern SMTZ was mainly distributed at 19–21 mbsf of the site SH3. The content of barium sulphate enrichment was apparent above the modern SMTZ which indicated that methane seepage during geological history was relatively stable and has lasted a long time, because the level of authigenic barium sulfate precipitation depended on the upward diffusion of dissolved Ba^2+^ flux and the length of time that the SMTZ remained at that horizon. By studying the trend of magnetic of sediments, the different type characteristic of carbonate mineral, results showed two stages of AOM (Figure 6). During the early AOM stage sulfate-driven AOM played a leading role (the main area of action in this paper is modern-SMTZ). The HS^-^ produced by sulfate-driven AOM reduced in situ metal oxides and provided a source of Fe^2+^ for formation of pyrite and other metal sulfides. Oxygen was gradually consumed during the dissimilatory reduction of Fe(III), so the sedimentary environment of the modern SMTZ gradually transformed from oxidizing environment convert to reducing environment. Meanwhile, the water produced by sulfate-driven AOM caused a decrease in pore water salinity. At the same time, most of the high-magnetic minerals (such as metal oxides) were converted into low-magnetic metal sulfides, which can cause a decrease in magnetic properties in the sediment during the SO_4_^2−^-AOM. However, there was no significant magnetic reduction in the modern SMTZ layer. Because it was in an oxidizing environment and Fe^2+^ was reoxidized to form new metal oxides. During the later AOM stage, metal-AOM was the source of Fe^2+^. The main area of action in this paper is paleo-SMTZ. A large number of metal oxides were converted into metal sulfides, causing significant magnetic decline. Nitrogen and phosphorus adsorbed on the surface of metal oxides were also released into the pore water, resulting in significant enrichment of pore water HPO_4_^−^. At the same time, due to the large amount of water produced by metal-AOM, pore water salinity was significantly reduced. The above features were observed in paleo-SMTZ indicating the presence of metal-AOM. Study of minerals in the paleo-SMTZ and modern SMTZ horizons showed that the SMTZ carbonate minerals in the current horizon were mainly low-Mg calcite and aragonite, while the paleo-SMTZ horizon carbon minerals were mainly high Mg. This was because the modern SMTZ layer was dominated by sulfate-driven methane anaerobic oxidation. In the paleo-SMTZ layer, the later metal-AOM stage primarily converted low-Mg carbonate minerals into high-Mg carbonate minerals.

## Appendix A

**Table A1:** Major and trace element contents in the sediments of core SH3.

Core Name	Depth (mbsf)	δ^34^S (‰-VCDT)	Al (%)	Ca/Ti	Sr/Ti	Fe (%)	Mn (%)	TOC (%)	Mo (ppm)	U (ppm)	Ba (ppm)
SH3B-1H	0.87		1.15	50.97	0.08	0.99	0.019	0.69	0.39	2.95	509.82
SH3B-1H	1.68	−44.8	7.31	25.23	0.11	3.46	0.042	0.92	0.47	4.64	465.70
SH3B-1H	2.57	−42.8	7.38	27.89	0.12	3.50	0.038	0.61	0.31	4.25	502.33
SH3B-1H	3.42	−46.5	8.18	13.01	0.06	3.88	0.033	1.11	0.97	4.32	437.91
SH3B-1H	4.27	−46.3	7.32	27.70	0.10	3.32	0.032	0.87	0.49	4.35	376.61
SH3B-1H	5.93	−41.8	6.93	37.71	0.13	3.05	0.033	0.71	0.36	4.61	396.00
SH3B-1H	6.22	−48.6	7.66	31.81	0.11	3.66	0.035	0.48	0.27	3.49	396.82
SH3B-1H	9.68	−36.0	7.46	29.79	0.11	3.61	0.039	0.43	2.14	6.37	450.18
SH3B-2H	10.57	−37.6	8.42	16.81	0.07	4.10	0.041	0.45	5.15	4.86	479.86
SH3B-2H	11.42	−39.2	8.02	24.00	0.10	3.68	0.038	0.47	1.50	4.38	459.43
SH3B-2H	13.12	−36.3	8.31	19.61	0.08	4.18	0.036	0.44	0.96	4.00	477.77
SH3B-2H	13.95	−33.3	7.56	31.27	0.12	3.62	0.041	0.34	0.68	2.86	446.76
SH3B-2H	14.82	−31.0	7.13	35.36	0.14	3.26	0.038	0.40	0.49	5.94	379.50
SH3B-2H	16.87	−30.2	8.01	18.92	0.07	3.45	0.034	0.54	0.84	4.08	407.07
SH3B-2H	17.68	−21.5	7.74	22.87	0.09	3.85	0.042	0.43	0.52	3.45	419.75
SH3B-2H	18.57	−13.3	6.98	34.83	0.12	3.56	0.039	0.31	0.34	2.60	453.02
SH3B-3H	19.42	−10.3	7.68	23.30	0.09	4.03	0.042	0.34	0.36	3.71	484.53
SH3B-3H	20.27	−12.7	8.56	13.91	0.07	4.17	0.050	0.39	0.47	3.14	454.82
SH3B-3H	21.08	−12.3	8.15	16.12	0.07	4.08	0.040	0.43	0.46	3.40	449.13
SH3B-3H	21.93	−12.2	7.77	21.89	0.09	4.01	0.040	0.38	0.85	3.42	509.35
SH3B-3H	22.82	−11.9	8.42	16.71	0.08	4.10	0.042	0.47	0.59	3.72	522.85

**Table A2:** Pore water contents in the sediments of core SH3.

Core Name	Depth (mbsf)	Water Sample ID	Salinity (ppt)	Sulfate (m)	Chlorinity (mM)	Chlorinity (mM)
SH3B-1H	0.85	1	35	21.6	562.1	562
SH3B-1H	1.66	2	35	26.1	551.6	552
SH3B-1H	2.55	3	35	25.8	560.8	561
SH3B-1H	3.4	4	35	25.5	559.2	559
SH3B-1H	4.25	5	34.5	23.4	555.9	556
SH3B-1H	5.06	6	34.5	21.9	555.4	555
SH3B-1H	5.91	7	34.5	21.3	549.3	549
SH3B-1H	6.2	8	34.5	21.4	557.3	557
SH3B-2H	8.85	9	33.5	14.6	558.8	559
SH3B-2H	9.66	10	33.5	13.4	558.3	558
SH3B-2H	10.55	11	33.5	12.4	559.2	559
SH3B-2H	11.4	12	33.5	11.5	558.3	558
SH3B-2H	12.25	13	33.5	11.1	559.7	560
SH3B-2H	13.1	14	33.5	10.5	556.4	556
SH3B-2H	13.91	15	33.5	9.8	555.4	555
SH3B-2H	14.8	16	33.5	9.8	557.3	557
SH3B-3H	16.85	17	33.1	9.4	559.2	559
SH3B-3H	17.66	18	33.1	8.4	560.7	561
SH3B-3H	18.55	19	32.6	7.1	557.3	557
SH3B-3H	19.4	20	33.1	6.3	558.8	559
SH3B-3H	20.25	21	32.6	4.8	558.8	559
SH3B-3H	21.06	22	33.1	3.5	557.3	557
SH3B-3H	21.91	23	32.6	0.6	558.3	558
SH3B-3H	22.8	24	32.6	3.6	558.3	558
